# The Effect of Ultraviolet Radiation on Bitumen Aging Depth

**DOI:** 10.3390/ma11050747

**Published:** 2018-05-07

**Authors:** Jinxuan Hu, Shaopeng Wu, Quantao Liu, Maria Inmaculada García Hernández, Wenbo Zeng, Shuai Nie, Jiuming Wan, Dong Zhang, Yuanyuan Li

**Affiliations:** State Key Laboratory of Silicate Materials for Architectures, Wuhan University of Technology, Wuhan 430070, China; hujinxuan221@whut.edu.cn (J.H.); liuqt@whut.edu.cn (Q.L.); maria.espersson@whut.edu.cn (M.I.G.H.); zwb0212@whut.edu.cn (W.Z.); nies1993@whut.edu.cn (S.N.); wanjm@whut.edu.cn (J.W.); pytmac@whut.edu.cn (D.Z.); liyuanyuan@whut.edu.cn (Y.L.)

**Keywords:** ultraviolet radiation, bitumen, aging depth, transmittance, permeation

## Abstract

The aging effect of ultraviolet (UV) radiation on bitumen has gained increasing attention from researchers, resulting in the emergence of a new method to simulate the UV aging that occurs during the service life of bitumen. However, the UV aging degree is closely related to bitumen thickness and the effect of UV radiation on aging depth is not clear. The relationship between ultraviolet (UV) radiation and bitumen UV aging depth was investigated in this paper. Three groups of samples were UV aged using different aging procedures to investigate the bitumen aging mechanism of UV radiation. The results from the first group showed that UV aging depth increased along with aging time. After aging for five hours, the complex modulus of the second and third layers increased. The second group’s results indicated that the aging effect of ozone was small and that the increase in aging depth was uncorrelated with ozone. The results from the third group showed that the transmittance of bitumen increased after UV aging and that the real reason why aging depth increased was permeation.

## 1. Introduction

Bitumen has been applied in the road construction of flexible pavement for many years. As a viscoelastic material, bitumen’s properties are closely related to many aspects of road performance [1]. However, because of external environmental effects such as oxygen [2] and ultraviolet (UV) radiation [3,4], bitumenious properties do not always satisfy operating requirements [5]. During the lifetime of pavement, bitumen experiences various aging processes. After the aging process, bitumen becomes harder and more brittle, which results in the degradation of pavement properties. Bitumen aging is a very complex process but the main cause is oxidation [6,7]. Oxidation results in consistency increasing and the volatile loss of bitumen [8]. The lifetime of pavement is reduced due to hard bitumen, especially under the heavy traffic conditions [9,10,11].

Bitumen aging generally consists of short-term thermal oxidation aging, long-term thermal aging and UV aging [12,13]. Although UV aging only occurs on superficial layers [14], the effect of UV aging on the properties of bitumen and pavement cannot be ignored [15,16]. Short-term thermal aging is generally evaluated using aging tests, such as the Thin Film Oven Test (TFOT, ASTM D 1754) or Rolling Thin Film Oven Test (RTFOT, ASTM D 2872) [17]. Long-term thermal aging is generally assessed by the Pressure Aging Test (PAV, ASTM D 6521) [18]. Unlike thermal aging, there is still no standard UV aging method for bitumen. After UV aging, the characterization methods for bituminous properties are still not unified [19,20].

The sample thicknesses adopted by some references are shown in Table 1. Sample thickness is an important parameter of UV aging methods, which is often ignored by researchers. Bitumen, after UV aging, may show different results if samples thicknesses are different. For example, UV radiation has been shown to have a significant effect on bitumen film with a thickness of 3 μm because of photochemical reactions. If the film thickness is thicker, the aging effect will be relatively smaller [21]. Katsuyuki Yamaguchi [22] used specimens with five different thicknesses of 50, 100, 200, 500 and 1000 μm to investigate the effect of film thickness on the UV aging of bitumen. The results showed that the thicker films had a lower elastic modulus, higher viscosity and relatively less production of carbonyl groups. The aging degree increased rapidly, especially when the film thickness was below 200 μm. Shaopeng Wu applied samples with four different film thicknesses (50, 100, 200 and 500 μm) to study the thermal, chemical and rheological properties of UV aged bitumen. The results indicated that the UV aging degree of bitumen was closely related to the film thickness of the sample.

From previous research, we know that UV radiation cannot influence the whole sample in a short time. Whether UV radiation influences bitumen at the bottom of samples where UV radiation cannot reach is still unknown. UV lamps release ozone during their runtime. The relationship between the aging effect on thin bitumen film and ozone is still unknown. Therefore, this paper describes a study investigating the relationship between UV radiation and UV aging depth. Three groups of samples were UV aged using different aging procedures to investigate the bitumen aging mechanism of UV radiation. Rheological properties and spectrum transmittance were tested to evaluate changes to bitumen properties.

## 2. Materials and Methods

### 2.1. Materials

The studied base bitumen was 60/80 penetration grade bitumen (B) obtained from KOCH Asphalt Co. Ltd. (Ezhou, Hubei Province, China). The physical properties of B are listed in Table 2.

### 2.2. Aging Procedure

Samples were exposed to UV radiation in a UV weathering oven (Fuzhou Meide Testing Instruments Co., Ltd, Fuzhou, China). The UV lamp was 500 W with a main wavelength of 365 nm. The aging procedures of different groups of samples were as follows.

**Figure 1:**
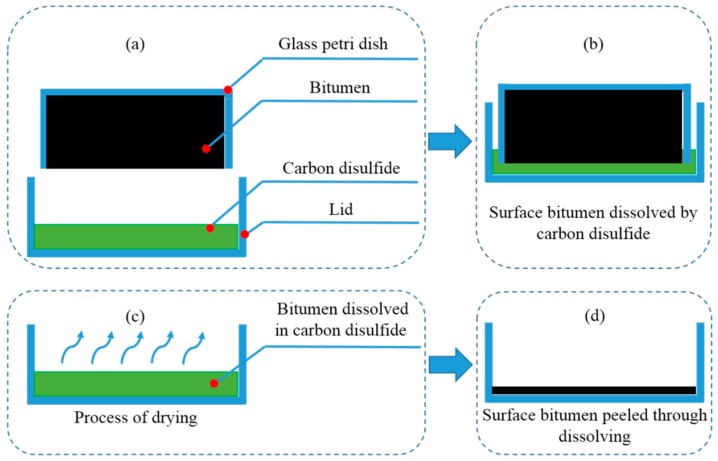
Peeling procedure, dissolving layer by layer.

**Figure 2:**
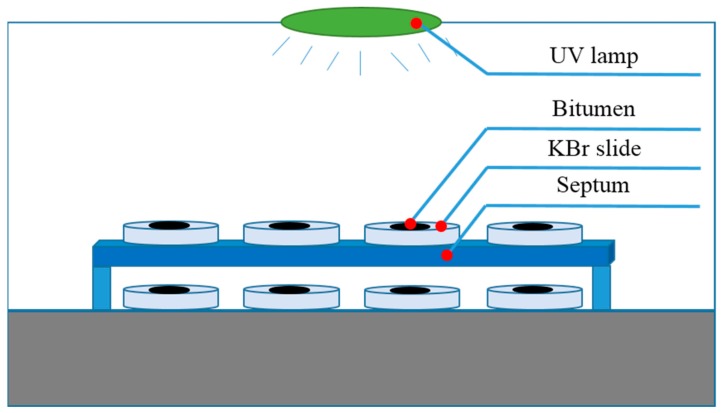
Samples were divided into two parts by septum.

**Figure 3:**
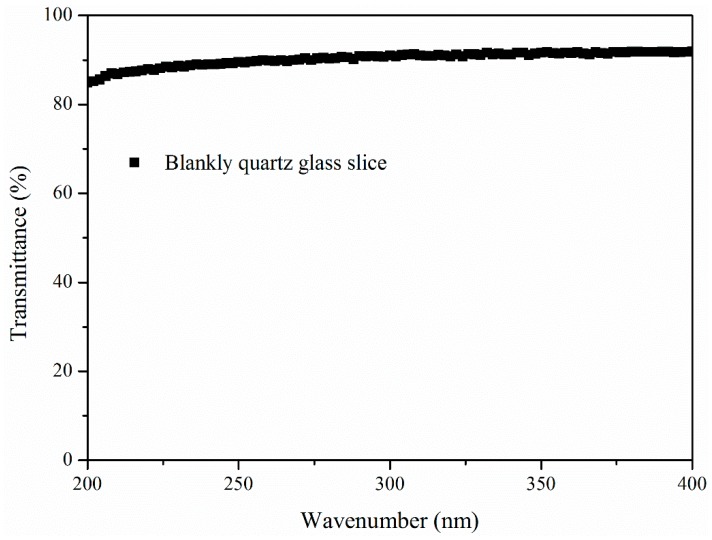
Transmittance of the blankly quartz glass slice with 1 mm thickness.

- The first group of samples was designed to investigate whether the aging depth would increase with aging time. Bitumen were poured into an Ø90 mm glass petri dish with its lid removed. The periphery of the dish was separated from the UV radiation by an insulating layer so that the UV radiation could only penetrate from the surface. Samples were UV aged for 1 h, 5 h and 10 h at 50 °C so that the UV aging procedure would not be affected by thermal oxidation aging [3]. The average intensity of UV radiation on the samples’ surface was 10 W/m^2^. The peeling procedure, dissolving layer by layer, is shown in Figure 1. After UV aging, bitumen on the surface was dissolved by carbon disulfide layer by layer (a) and (b). The dissolved bitumen for one layer was almost 0.8 g and the thickness of the layer was approximately 105 μm. Then the solutions were dried for 7 d (c) and the residues (d) were tested by DSR. Every sample was dissolved three times. The first layer means that the layer was dissolved at the beginning and the third layer means that the layer was dissolved at the end. The first, second and third layers of B aged by UV for 1 h were abbreviated as B-1h-1, B-1h-2 and B-1h-3.
- The second group of samples was used to explore the aging effect between samples with and without UV radiation. Bitumen was dissolved by carbon disulfide first and then the solutions were dropped onto a blank KBr slide [29,30]. After drying, the solutions turned into a thin film. The concentration of the solutions was 5 wt % and the thickness of the film after drying was approximately 10 μm. Samples were divided into two parts, as shown in Figure 2. These parts were separated by a septum, which UV radiation cannot penetrate. The region between the septum and the samples below the septum was approximately 1 cm, which guaranteed that the atmosphere could circulate. Samples were UV aged for 5 d at 50 °C. The average intensity of UV radiation on the Bu’ surface was 10 W/m^2^. The ozone concentration was about 0.4 ppm in the environment and about 1.2 ppm in the oven, which was tested by a gas detector (uSafe 2000/300, Sundo Shenzhen Technology Co., Ltd., Shenzhen, China). After UV aging, samples were tested by Fourier Transform Infrared (FTIR, Nexus, Thermo Nicolet Corp., Waltham, MA, USA) Spectroscopy. Samples on the septum and samples below the septum were abbreviated as Bu and Bb respectively.
- The third and last group of samples was used to study the UV transmittance of bitumen with aging time. Bitumen was dissolved by carbon disulfide, then solutions with different concentrations (2.5%, 5%, 10%, 15%, 20%, and 30%) were dropped into a quartz glass slice, respectively, and different thicknesses (1.1 μm, 2.3 μm, 5.6 μm, 6.3 μm, 8.1 μm, and 13.1 μm) were obtained. Then, the slice was put on the spin coater and ultrathin bitumimous film was prepared. Samples were UV aged for 0 h, 1 h, 5 h, 10 h, or 50 h. Transmittance of the blankly quartz glass slice with 1 mm thickness is shown in Figure 3. The UV radiation transmittance of the slice from 200 nm to 400 nm was larger than 85%. It was considered that the slice would not influence the UV radiation transmittance in this paper.

### 2.3. Characterization Method

#### 2.3.1. Dynamic Shear Rheometer (DSR)

The rheological properties of the bitumen samples were tested by DSR (MCR101, Anton Paar Corp., Graz, Austria) under strain-controlled mode. High temperature sweep tests were adopted, with temperatures ranging from 30 °C to 60 °C. The constant frequency was 10 rad/s and the temperature increment was 2 °C per minute. The diameter of the plate was 25 mm and the gap between the plates was 1 mm. Essential rheological parameters such as complex modulus (G*) and phase angle (δ) can be obtained from a DSR test.

#### 2.3.2. Fourier Transform Infrared (FTIR) Spectroscopy

FTIR test was performed under transmission mode, which provides the transmittance of the bitumen under each wavelength. The spectra of the bitumen ranging from 4000 cm^−1^ to 400 cm^−1^ can be obtained from FTIR (Nexus, Thermo Nicolet Corp., Waltham, MA, USA). The spectral resolution was 4 cm^−1^. Chemical structures can be distinguished from the spectra, which are related to different chemical bonds. From the FTIR test, the sulfoxide group S=O (centered around 1030 cm^−1^) and the carbonyl group C=O (centered around 1700 cm^−1^) were monitored by studying their spectra changes. From the carbonyl group and sulfoxide group, information on the oxidation of the asphalt could be characterized. Through the area of their bonds in the spectra, the sulfoxide group S=O index (I_S=O_) and carbonyl group C=O index (I_C=O_) could be calculated using the following equations [31,32], which were used to characterize the aging degree:

(1) $$I_{S=O}=\frac{\mathrm{Area}\;\mathrm{of}\;\mathrm{sulphoxide}\;\mathrm{band}\;\mathrm{centered}\;\mathrm{around}1030{\;\mathrm{cm}}^{-1}}{\sum \mathrm{Area}\;\mathrm{of}\;\mathrm{spectral}\;\mathrm{bands}\;\mathrm{between}\;2000\;\mathrm{and}\;600{\;\mathrm{cm}}^{-1}}$$

(2) $$I_{C=O}=\frac{\mathrm{Area}\;\mathrm{of}\;\mathrm{carbonyl}\;\mathrm{band}\;\mathrm{centered}\;\mathrm{around}\;1700{\;\mathrm{cm}}^{-1}}{\sum \mathrm{Area}\;\mathrm{of}\;\mathrm{spectral}\;\mathrm{bands}\;\mathrm{between}\;2000\;\mathrm{and}\;600{\;\mathrm{cm}}^{-1}}$$

#### 2.3.3. Ultraviolet Spectrophotometry

Transmittance of the third group was tested by UV spectrophotometry (Lambda 750S, PerkinElmer, Waltham, MA, USA). The wavelength scan ranged from 200–800 nm and the average spectrum was calculated. UV radiation with different wavelengths can be absorbed by bitumen because of its chemical bonds and transmittance may be reduced. After UV aging, the chemical bonds change, which results in changes in transmittance. Consequently, the transmittance tested by UV spectrophotometry could be used to characterize the aging degree.

## 3. Results and Discussion

### 3.1. Aging Effect on Bitumen in Different Layers for Different Aging Times

#### 3.1.1. Complex Modulus of Bitumen in Different Layers at High Temperatures

**Figure 4:**
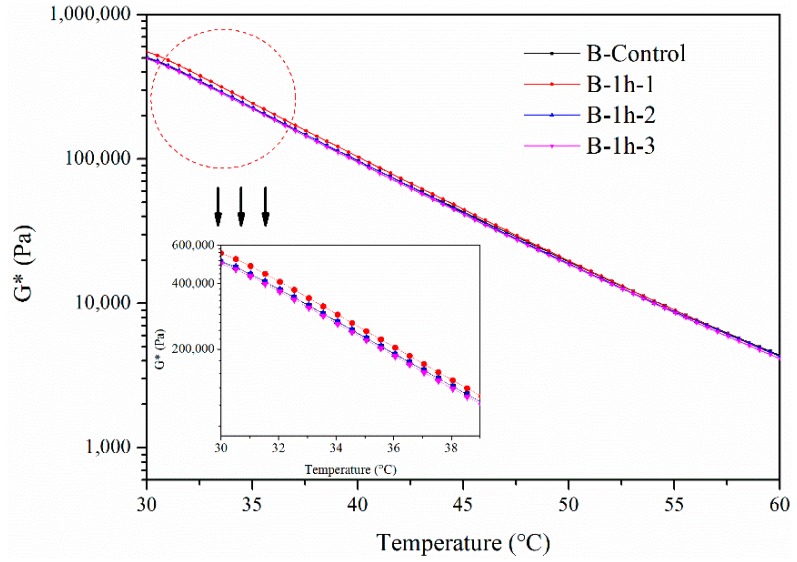
Complex modulus of B aged by UV radiation for 1 h.

The complex modulus of B in different layers for 1 h from 30 °C to 60 °C is shown in Figure 4. The complex modulus of B in the first layer slightly increased after aging for one hour. This means that it was influenced by the UV radiation. Furthermore, the complex modulus of B in the second and third layers was the same as complex modulus of the original bitumen. This means that the UV radiation had no aging effect on the bitumen in these two layers.

**Figure 5:**
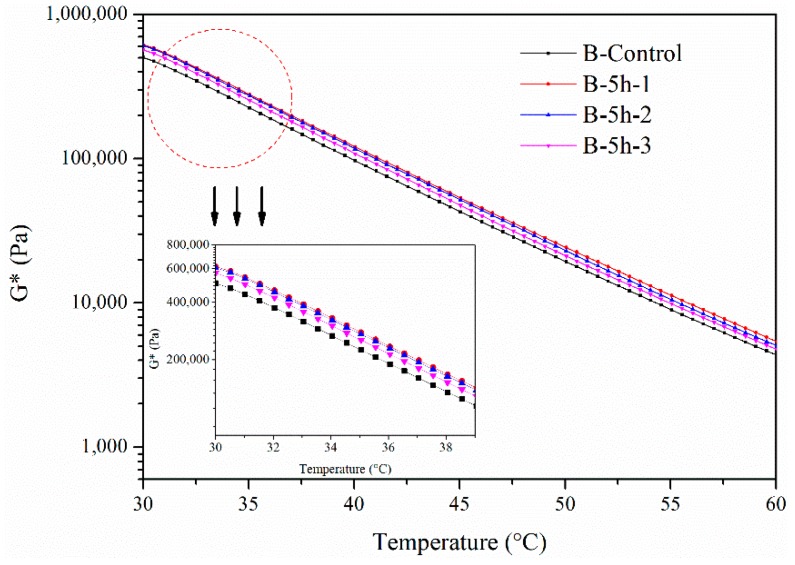
Complex modulus of B aged by UV radiation for 5 h.

The complex modulus of B in different layers for 5 h from 30 °C to 60 °C is shown in Figure 5. The complex modulus of B in the first and the second layers obviously increased after aging. The complex modulus of B in the third layer was a little higher than the complex modulus of the original bitumen. This means that after aging for 5 h, B in the second and the third layers began to be aged by the UV radiation.

**Figure 6:**
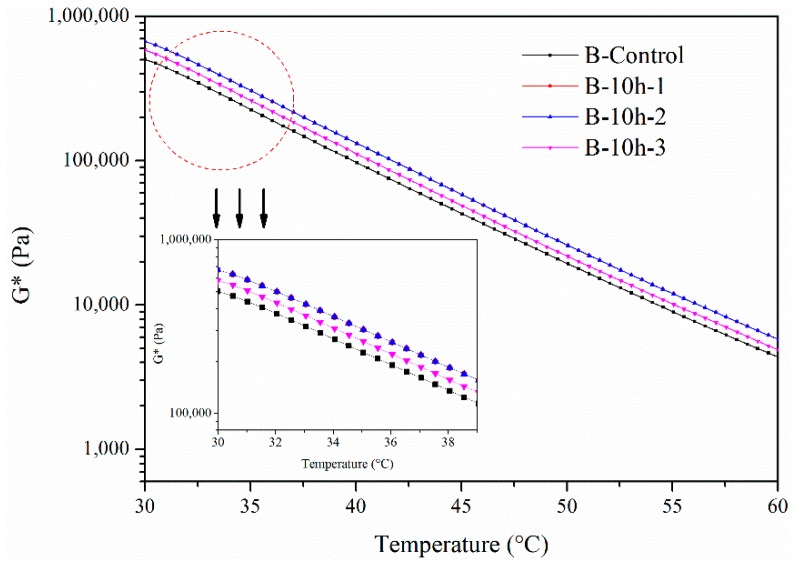
Complex modulus of B aged by UV radiation for 10 h.

**Figure 7:**
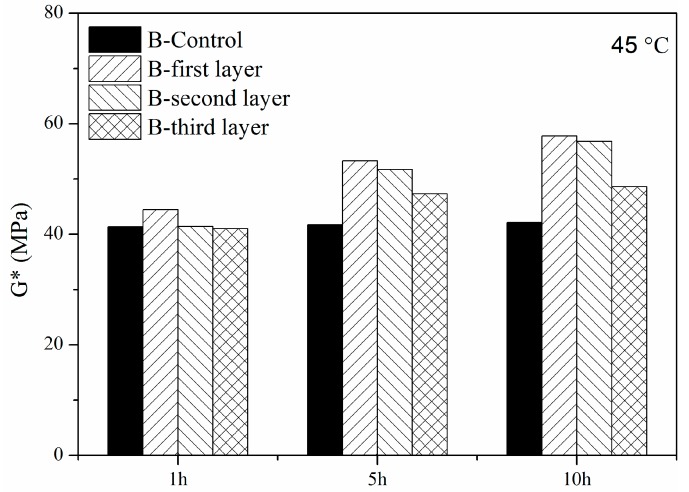
Complex modulus (45 °C) of B in different layers for different aging times.

The complex modulus of B in different layers for 10 h from 30 °C to 60 °C is shown in Figure 6. Furthermore, the complex modulus of bitumen at 45 °C in different layers is illustrated in Figure 7. The tendency of the UV aging of the bitumen was similar to the situation in which the bitumen was aged for 5 h. The complex modulus of B in these three layers was higher than it was in the original bitumen. This means that UV radiation influenced B in these three layers after aging for 10 h. Additionally, B in the first and the second layers showed a higher complex modulus than in the third layer, which means that the aging effect in different layers was different.

#### 3.1.2. Complex Modulus of Bitumen in the Third Layer for Different Aging Times

**Figure 8:**
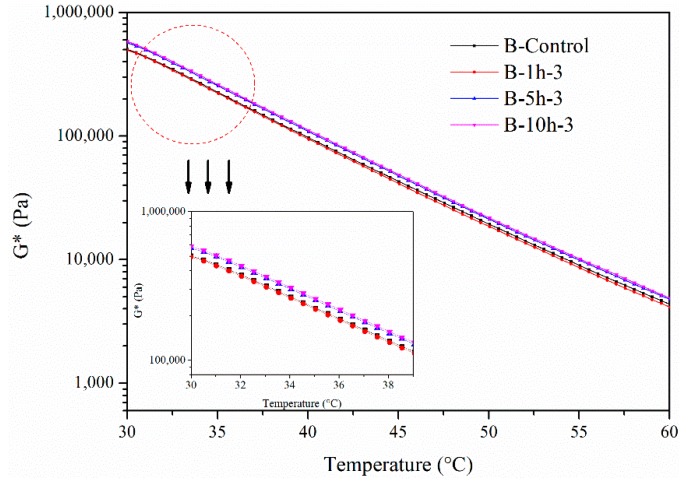
Complex modulus of B in the third layer’s bitumen for different aging times.

The complex modulus of B in the third layer, aged for different aging times from 30 °C to 60 °C, is shown in Figure 8. It can be seen that the complex modulus of B in the third layer aged for 1 h was the same as the original bitumen. After aging for 5 h, the complex modulus increased. The degree of aging was accelerated after aging for 10 h. This means that UV radiation only had an impact on the surface bitumen. With an increase in aging time, the aging depth due to UV radiation reached the first layer after 1 h and after UV aging for 10 h, the aging depth increased and was no longer in the first layer. In other words, aging depth accelerated due to changes in material properties.

### 3.2. Aging Effect on the Second Group Caused by Ozone

**Figure 9:**
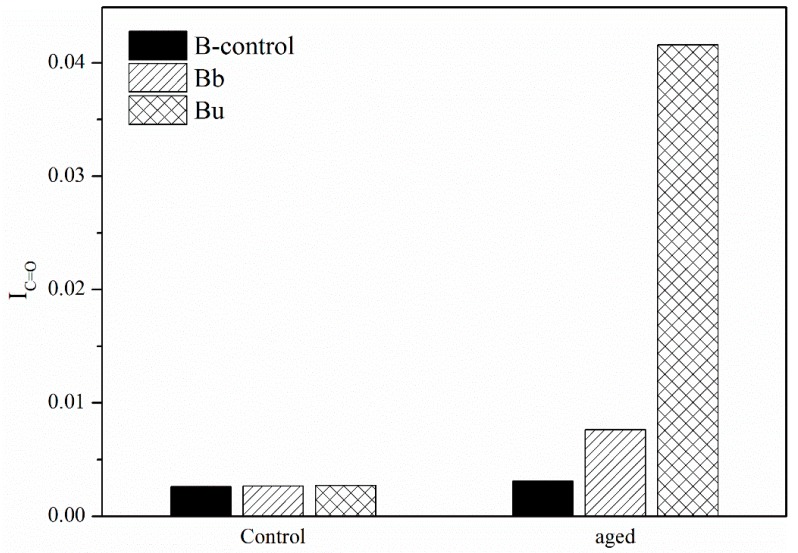
I_C=O_ of the second group before and after UV aging.

**Figure 10:**
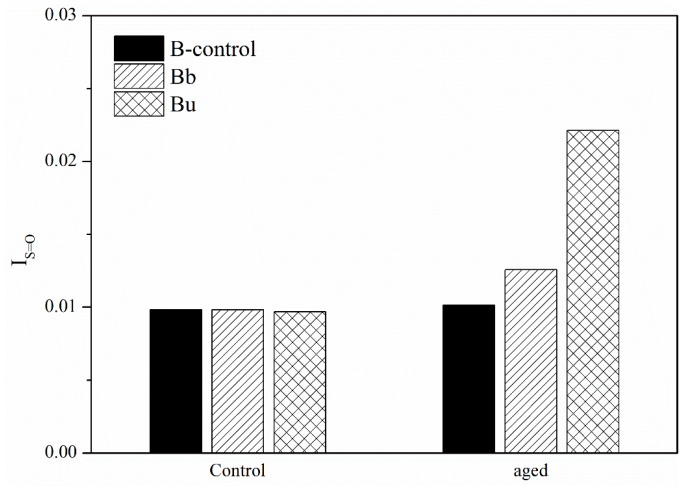
I_S=O_ of the second group before and after UV aging.

I_C=O_ and I_S=O_ of B in the second group are shown in Figure 9 and Figure 10. After UV aging, I_C=O_ and I_S=O_ of Bu increased. However, I_C=O_ and I_S=O_ of Bb was only slightly more than original bitumen. This means that the bitumen on the septum was seriously aged by the UV radiation. Otherwise, the bitumen below the septum was influenced by ozone and the effect was very small. Furthermore, although ozone had an effect on bitumen, the increment of aging depth was not closely related to ozone because the effect was too small.

### 3.3. Transmittance of Bitumen after UV Aging

#### 3.3.1. Transmittance of Bitumen with Different Thicknesses after UV Aging

**Figure 11:**
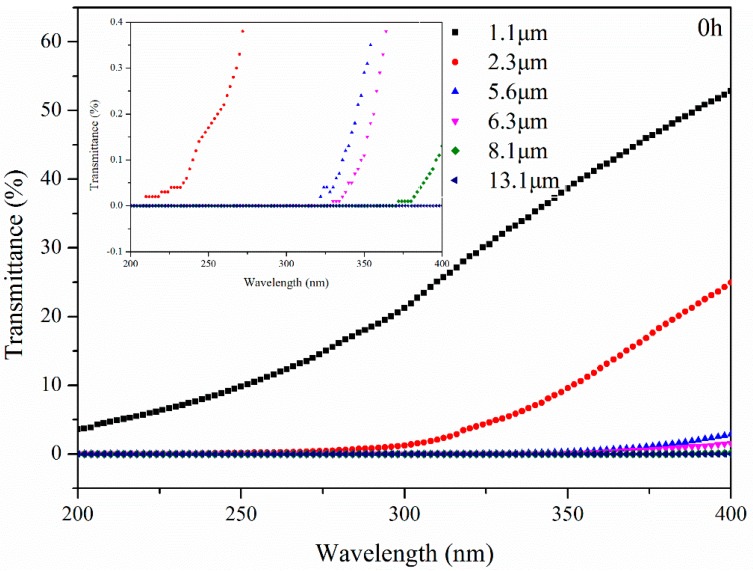
Transmittance of bitumen with different thicknesses after UV aging for 0 h.

**Figure 12:**
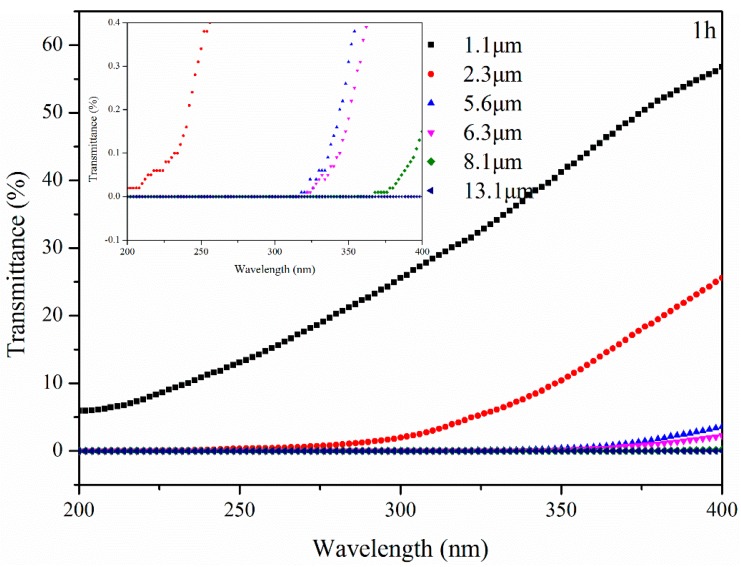
Transmittance of bitumen with different thickness after UV aging for 1 h.

The transmittance of bitumen with different thicknesses after UV aging for 0 h is displayed in Figure 11. From Figure 12, it can be seen that the transmittance of B gradually decreased with the increase in bitumen thickness. This can be explained by the thicker bitumen film blocking light radiation more effectively. Meanwhile, transmittance gradually increased with the increase in wavelength. This means that the transmittance of the UV radiation at different wavelengths was different in this wavelength scope. Furthermore, as shown in Figure 10, the transmittance of B with 1.1 μm, 2.3 μm, 5.6 μm, 6.3 μm, 8.1 μm, and 13.1 μm thickness was 52.79%, 24.96%, 2.85%, 1.57%, 0.13%, and 0% at 400 nm, respectively. This means that the transmittance of UV radiation is very limited and that B with 13.1 μm thickness could block all UV radiation. Additionally, because of the different transmittance of different wavelength UV radiation, B with 2.3 μm, 5.6 μm, 6.3 μm and 8.1 μm thickness blocked UV radiation at the 210 nm, 322 nm, 330 nm, 372 nm wavelengths, respectively. UV radiation from 200 nm to 400 nm cannot blocked by B with 1.1 μm thickness.

Figure 11 shows the transmittance of bitumen with different thicknesses after UV aging for 1 h. After UV aging, the transmittance of B with different thicknesses increased. All the UV radiation from 200 nm to 400 nm could pass through B with 2.3 μm thickness. UV radiation was blocked at 318 nm, 322 nm, and 368 nm by B with 5.6 μm, 6.3 μm and 8.1 μm thickness, respectively. The transmittance of B with 13.1 μm was still 0%. This means that UV radiation still cannot pass through a bitumen film with 13.1 μm thickness, although the transmittance of B increased because of the UV radiation.

**Figure 13:**
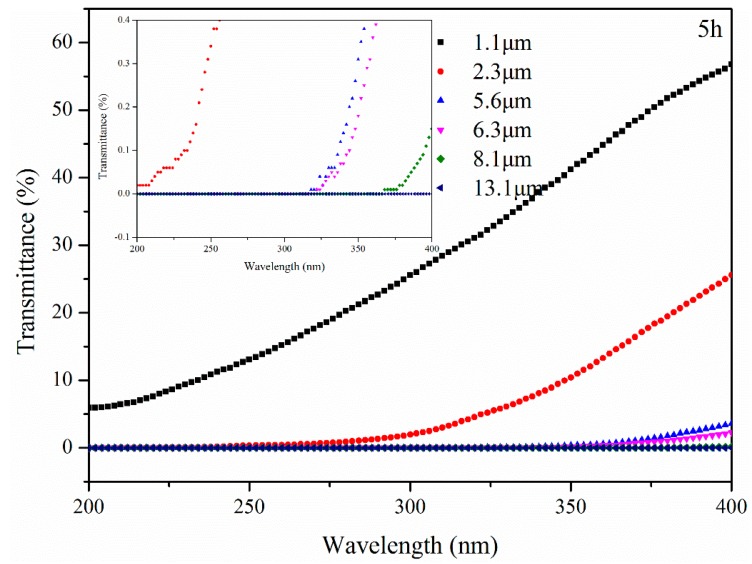
Transmittance of bitumen with different thickness after UV aging for 5 h.

The transmittance of bitumen with different thicknesses after UV aging for 5 h is shown in Figure 13. The transmittance of B with 1.1 μm, 2.3 μm, 5.6 μm, 6.3 μm and 8.1 μm thickness at 400 nm increased to 63.74%, 25.66%, 3.92%, 2.46% and 0.45%, respectively. However, UV radiation was still blocked by B with 13.1 μm thickness.

**Figure 14:**
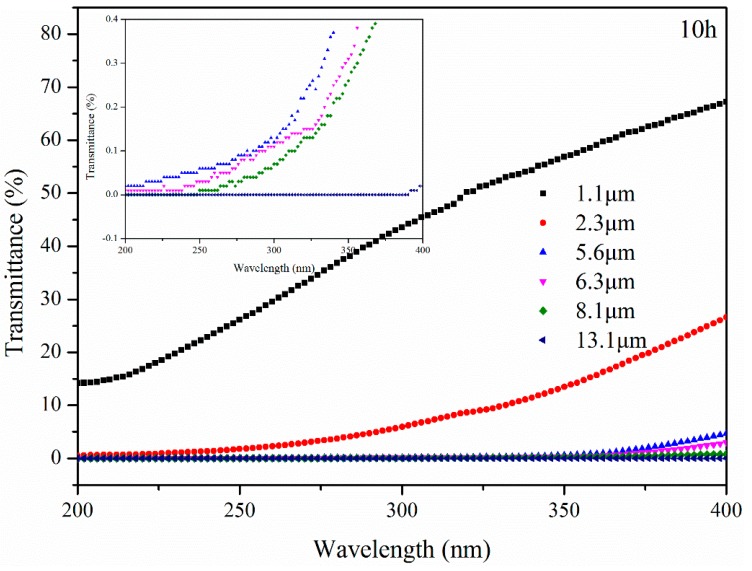
Transmittance of bitumen with different thickness after UV aging for 10 h.

The transmittance of bitumen with different thicknesses after UV aging for 10 h is illustrated in Figure 14. The transmittance of B further increased after UV aging. Furthermore, UV radiation from 200 nm to 400 nm wavelength could pass through B with 5.6 μm and 6.3 μm thickness after aging for 10 h. This means that after aging for 10 h, bitumen below 6.3 μm thickness of surface was aged by UV radiation at all wavelengths directly. Otherwise, B with 8.1 μm and 13.1 μm thickness blocked UV radiation at the 250 nm and 392 nm wavelengths, respectively. This means that B below 13.1 μm thickness began to be influenced by UV radiation from 392 nm to 400 nm directly and that the effect was extremely small.

**Figure 15:**
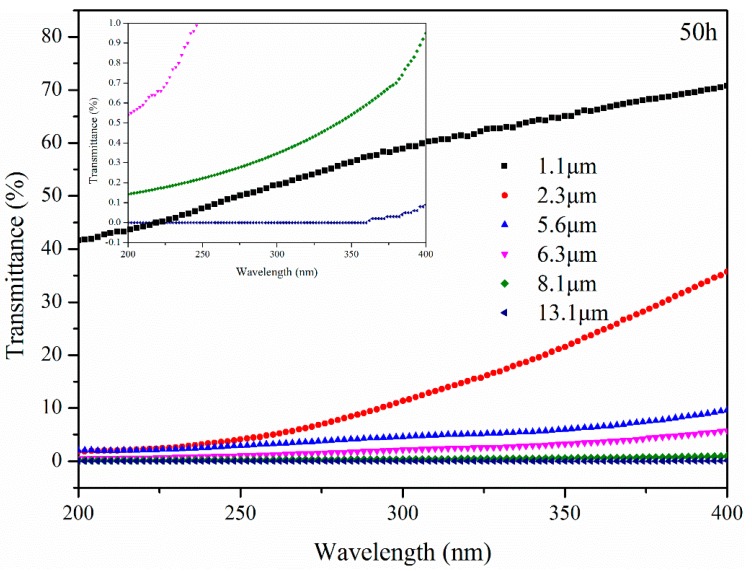
Transmittance of bitumen with different thickness after UV aging for 50 h.

Figure 15 presents the transmittance of bitumen with different thicknesses after UV aging for 50 h. After aging for 50 h, UV radiation from 200 nm to 400 nm could pass through B with different thicknesses, except for 13.1 μm thickness. This means that B under a surface of 8.1 μm thickness was aged by UV radiation directly. UV radiation was blocked at 362 nm by B with 13.1 μm thickness. B below 13.1 μm thickness could be aged by more UV radiation compared to B that was aged for 10 h.

#### 3.3.2. UV Aging Model

**Figure 16:**
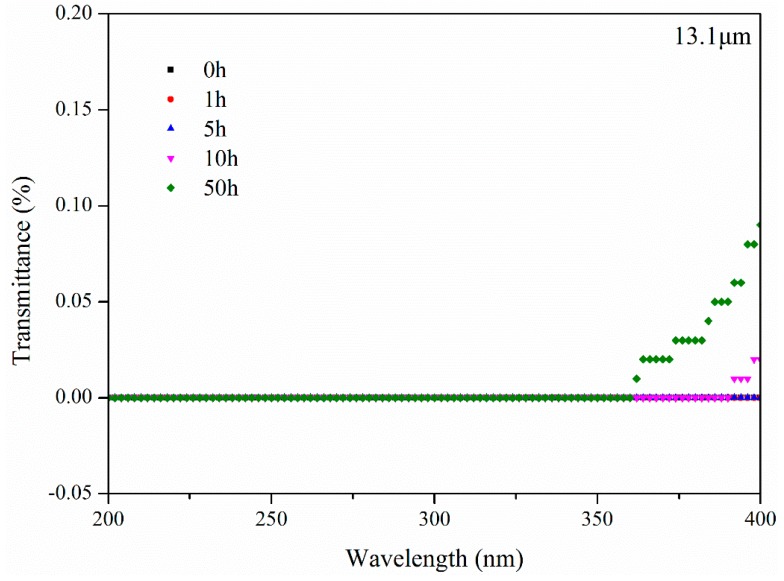
Transmittance of bitumen with 13.1 μm thickness after UV aging for different aging times.

**Figure 17:**
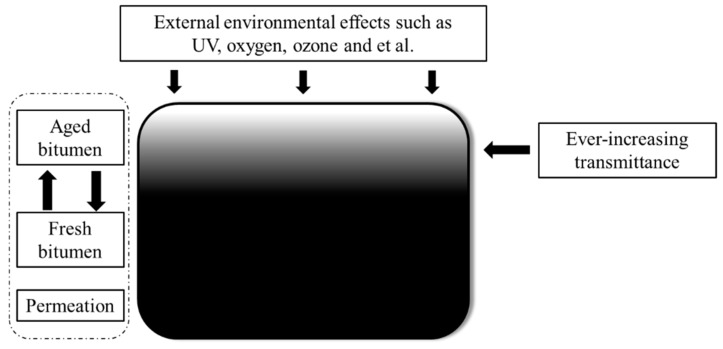
UV aging model.

The transmittance of bitumen with 13.1 μm thickness after UV aging for different aging times is illuminated in Figure 16. It was observed that UV radiation could not reach a depth greater than 13.1 μm thickness below the bitumen surface before aging for 5 h. As the aging time increased, UV radiation reached deeper. After aging for 10 h, sectional UV radiation could pass through bitumen with 13.1 μm thickness. However, from the results of the first group, it was clear that there was a UV aging effect on bitumen after aging for 5 h. This means that bitumen which had no direct contact with UV radiation was influenced by UV radiation. Furthermore, external environmental effects should be excluded according to the results of the second group. The critical point of this phenomenon is that bitumen which had no direct contact with UV radiation, had direct contact with bitumen aged by UV radiation. The real reason why bitumen which had no direct contact with UV radiation was influenced by UV radiation, is permeation. Aged bitumen permeates fresh bitumen and fresh bitumen permeates aged bitumen. The same tests were also conducted on another bitumen sample (80/100 penetration grade) and very similar results and the same conclusions were obtained. The UV aging effect is explained in Figure 17. Bitumen on the surface was subjected to various external environmental effects such as ozone and UV radiation. Bitumen at the bottom encountered the effects of permeation and ever-increasing UV radiation transmittance.

## 4. Conclusions

The effect of UV radiation on bitumen aging depth was investigated. The rheological properties and FTIR results of three groups of samples were analyzed. The following conclusions can be drawn based on the results:

1. After aging for 5 h, bitumen in the second and the third layers began to be aged by UV radiation. As aging time increased, aging depth increased.
2. The aging effects of ozone were small. Bitumen is hard age if it has no contact with UV radiation.
3. The UV radiation transmittance of bitumen increased after UV aging. After UV aging for 50 h, partial UV radiation could pass through bitumen with 13.1 μm thickness.
4. After aging for 5 h, UV radiation from 200 nm to 400 nm still could not pass through bitumen with 13.1 μm thickness. The real reason why aging depth increased was permeation. Aged bitumen permeated fresh bitumen, which resulted in changes to the rheological properties of the second and third layers.
5. Permeation is closely related to the thermal stability, therefore, bitumen on road surfaces should possess high thermal stability to ensure its performance.

## Figures and Tables

**Table 1:** UV aging conditions and sample thicknesses of previous research.

Author	UV Aging Conditions	Sample Thickness	References
Virginie Mouillet	Temperature: 60 °C Aging time: 60 h	Sample thickness: 10 μm	[23]
Franҫoise Durrieu	Temperature: 60 °C Aging time: 170 h	Sample thickness: 10 μm	[16]
Xinyu Zhao	Temperature: 60 °C Aging time: 72 h	Sample thickness: 0.5 mm	[13]
de Sá Araujo	Temperature: 60 °C Aging time: 10, 20, 30, 40, 50, 100, 150 and 200 h	Sample thickness: 0.6 mm	[24]
Henglong Zhang	Temperature: 60 °C Aging time: 12 d	Sample thickness: 1.92 mm	[25]
Zhengang Feng	Temperature: 60 °C Aging time: 6 d	Sample thickness: 2 mm	[26]
Peiliang Cong	Temperature: 60 °C Aging time: 7 d	Sample thickness: 3 mm	[27]
Song Xu	Temperature: 60 °C Aging time: 9 d	Sample thickness: 3.2 mm	[28]

**Table 2:** Physical properties of B.

Physical Properties	B
Softening point (°C)	49.0
Penetration (25 °C, 0.1 mm)	77.5
Ductility (5 °C, 1 cm/min)	8.9
Viscosity (60 °C, Pa s)	205
Viscosity (135 °C, Pa s)	0.46
